# Spontaneous Regression of Clear Cell Renal Cell Carcinoma Metastases or Immune Restoration?

**DOI:** 10.3390/curroncol33050282

**Published:** 2026-05-10

**Authors:** Clara Vacheret, Fabien Moinard-Butot, Lucile Reberol, Alexandre Ciccolini, Roberto Luigi Cazzato, Philippe Barthélémy

**Affiliations:** 1 Department of Medical Oncology, University Hospital of Strasbourg, 67200 Strasbourg, France; clara.vacheret@aphp.fr (C.V.); lucile.reberol@chru-strasbourg.fr (L.R.); alexandre.ciccolini@chru-strasbourg.fr (A.C.); philippe.barthelemy@chru-strasbourg.fr (P.B.); 2 Department of Interventional Radiology, University Hospital of Strasbourg, 67000 Strasbourg, France; robertoluigi.cazzato@chru-strasbourg.fr

**Keywords:** spontaneous regression, clear cell renal cell carcinoma, cryoablation, abscopal effect, immune reconstitution

## Abstract

Spontaneous regression of metastatic renal cell carcinoma is a rare and incompletely understood phenomenon. We report the case of a 61-year-old man with biopsy-proven pulmonary metastases from clear cell renal cell carcinoma who experienced durable tumor regression without receiving any systemic therapy. The patient underwent cryoablation of a symptomatic iliac bone metastasis and discontinued methotrexate, previously prescribed for inflammatory polyarthritis. Serial imaging demonstrated initial slow progression followed by significant shrinkage of pulmonary and mediastinal lesions, leading to a sustained partial response according to RECIST 1.1 criteria. No disease progression has been observed after extended follow-up. Two non-mutually exclusive mechanisms may explain this observation: restoration of antitumor immunity following withdrawal of immunosuppressive therapy, and a systemic immune response triggered by local tumor destruction (abscopal effect). Although such events are exceptional, this case highlights the potential interplay between immune modulation and local therapies in renal cell carcinoma. Further investigation is warranted to better understand these mechanisms and their potential therapeutic implications.

## Simple Summary

Spontaneous regression of cancer is a rare and poorly understood phenomenon, particularly in metastatic renal cell carcinoma. We report the case of a patient whose lung metastases regressed without any systemic treatment after local therapy of a bone lesion and discontinuation of an immunosuppressive drug. Two main hypotheses may explain this observation: restoration of the immune system after stopping immunosuppression, or a systemic immune response triggered by local tumor destruction (known as an abscopal effect). Such cases remain exceptional, especially without immunotherapy. This report highlights the potential role of the immune system in controlling cancer progression and suggests that interactions between local treatments and immune responses deserve further investigation.

## 1. Introduction

Spontaneous tumor regression has been reported since the mid-twentieth century, and is the subject of much speculation and hypothesis as to the underlying mechanisms [1]. Spontaneous tumor regression is defined as the partial or complete disappearance of a malignant tumor without any therapy known to induce an antineoplastic effect [2]. A first systemic review of 176 cases documented in the literature was reported in 1966 by Everson and Seligman, then in 1990 by Challis, who listed 504 cases published between 1900 and 1987 [3]. Renal carcinomas account for 20–25% of spontaneous regression cases, but histological confirmation is described in only 20% of cases [4,5,6,7,8,9]. In the majority of cases, the site of metastatic regression is the lung, and this occurs after nephrectomy [10].

Several hypotheses have been proposed, including immune reactivation following tumor debulking, infection-induced immune stimulation, withdrawal of immunosuppressive therapies, and systemic immune responses triggered by local treatments [11]. The latter mechanism, known as the abscopal effect, has been primarily described in association with radiotherapy and, more recently, in combination with immunotherapy. Reports of abscopal-like effects following cryoablation remain exceedingly rare, particularly in patients not receiving systemic immune-modulating agents.

We report here the case of a patient with metastatic renal clear cell carcinoma (ccRCC) who experienced a “spontaneous” regression of multiple pulmonary and lymph node metastases without any systemic therapy following local treatment consisting of nephrectomy and cryoablation of a single symptomatic bone metastasis, as well as the discontinuation of methotrexate (MTX), an immunosuppressive agent.

## 2. Case Presentation

Our patient is a 61-year-old man at the time of initial diagnosis. His medical history includes arterial hypertension on dual therapy, metabolic syndrome, sleep apnea syndrome, non-insulin-dependent diabetes mellitus on Metformin and a DDP4 inhibitor, dyslipidemia, former smoker, pulmonary silicosis secondary to occupational exposure (coal mine worker) and rheumatoid-like polyarthritis treated with an immunosuppressive therapy (methotrexate 15 mg/week). Multiple chest CT scans performed between 2015 and 2022 for follow-up of pulmonary silicosis showed no evidence of pulmonary nodules or lymph node enlargement prior to October 2022.

**Figure 1:**
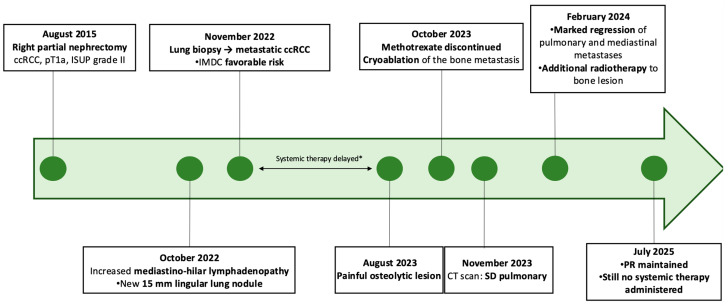
Clinical timeline evolution and treatment ccRCC. ccRCC: clear cell renal carcinoma; SD: stable disease; PR: partial response.

In 2015, a right renal mass was detected incidentally on a CT scan performed to assess inflammatory rheumatism (Figure 1). The CT scan, as part of the extension work-up, found no distant metastases, but described a mediastino-axillary polyadenomegaly of unclear significance. A right partial nephrectomy was performed in August 2015, confirming an ISUP grade II ccRCC, classified as stage pT1a Nx, with negative surgical margins. Biannual follow-up with CT scanning was implemented.

**Figure 2:**
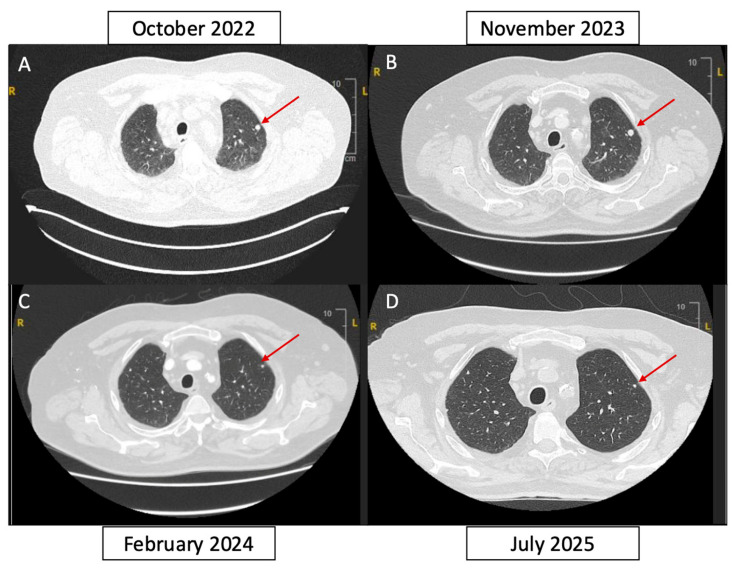
Radiological evolution of pulmonary metastases between 2022 and 2025 in the absence of systemic therapy. (**A**) Chest CT scan from October 2022 showing a 9 mm subpleural pulmonary nodule. (**B**) November 2023 CT scan demonstrating slow progression of pulmonary metastases prior to methotrexate discontinuation and cryoablation of the symptomatic bone lesion. (**C**) February 2024 CT scan revealing spontaneous regression with marked reduction in the size of pulmonary metastases (subpleural nodule: 9 mm → 2 mm). (**D**) July 2025 CT scan confirming continued tumor regression and a durable partial response according to RECIST 1.1. The red arrow indicates the lung tumor.

In October 2022, a thoracic CT scan performed as part of the follow-up for pulmonary silicosis revealed an increase in the size of mediastino-hilar lymph nodes and the appearance of a lingular lung tumor of 15 mm and right subpleural tumor of 9 mm in size (Figure 2). A lung biopsy was performed in November 2022 and confirmed the metastasis of a ccRCC, presumed ISUP 2, that was TFE3-negative with no sarcomatoid component. The patient belonged to the IMDC favorable risk group (Figure 1).

The onset of systemic treatment was delayed due to the patient’s heavy cardiovascular history, which temporarily contraindicated anti-angiogenic therapy, and polyarthritis on immunosuppressive therapy, which contraindicated the initiation of immunotherapy. In view of the slow progression of pulmonary lesions, we recommend managing cardiovascular risk factors in cardiology, and performing dental treatment prior to initiation of anti-angiogenic therapy. CT scan monitoring continued for several months, and the disease progressed slowly (Figure 2).

In August 2023, an osteolytic lesion of the right iliac wing appeared, accompanied by pain and functional impotence. The patient presented with opposite pain and functional impotence. The bone lesions did not undergo histologic sampling but were considered metastatic based on their radiologic characteristics, clinical evolution, and concordant timing with disease progression. In view of the oligo-metastatic evolution of the ccRCC, we scheduled local treatment with cryoablation and we discussed with his rheumatologist stopping immunosuppressive therapy before introducing systemic treatment. On October 2023, the patient discontinued the MTX therapy (Figure 1).

At the same time, the patient underwent cryotherapy with four Icerod needles placed inside the lesion and two through the vertebroplasty trocars. After peri-lesional hydrodissection, two cryotherapy cycles of 10 and 8 min were performed (100%). At the end of the procedure, two screws were inserted through the trocars and filled with Osteopal cement (Figure 1).

We met the patient in December 2023; he had fully recovered and the analgesics had been weaned. Discontinuation of MTX had not led to a resumption of polyarthritic activity. The CT scan performed in November showed the overall stable disease of all lung metastases. We recommended that the patient underwent the necessary dental treatment before the introduction of anti-angiogenic therapy in the event of frank disease progression.

In February 2024, the CT scan showed a clear tumor shrinkage of mediastinal lymph nodes and lung metastases, suggesting spontaneous regression. The right apex nodule was reduced from 6 mm to 5 mm compared with the August 2023 scan, the subpleural nodule from 9 mm to 2 mm (Figure 2), and the metastasis in the lower segment of the lingula from 14 mm to 5 mm. Close monitoring was maintained. At the same time, the patient received additional radiotherapy on the bone lesion treated by cryoablation, with a total dose of 35 Gy in five fractions of 7 Gy (Figure 1).

In February 2026, the CT scan confirmed partial response according to RECIST 1.1 criteria, with the patient maintaining a durable response more than two years after the initial detection of lung metastases and without any systemic therapy (Figure 2). At the most recent follow-up in July 2025, the cryoablated iliac lesion remained stable, with sclerosis consistent with post-ablative changes and no evidence of local recurrence.

## 3. Discussion

Spontaneous regression of mRCC is a rare but well-documented phenomenon. Although its underlying mechanisms remain uncertain, most hypotheses converge on the restoration or activation of an effective antitumor immune response [12,13]. In the present case, several immunologically relevant events occurred in close temporal association with the regression of metastatic lesions, raising the possibility of converging or synergistic mechanisms. Low-dose MTX, commonly used in inflammatory rheumatologic diseases, exerts systemic immunosuppressive effects by inhibiting lymphocyte proliferation and impairing T-cell-mediated immunity [14]. Immune reconstitution phenomena have been observed after MTX discontinuation, including cases of MTX-associated lymphoproliferative disorders regressing upon treatment withdrawal [14,15]. In our patient, the cessation of MTX preceded the radiologic tumor regression by several months and did not result in a flare of the underlying polyarthritis, suggesting a rapid and effective immune reconstitution. The recovery of T-cell function could have allowed reactivation of antitumor immune surveillance, thereby contributing to the progressive shrinkage of metastases. Although this mechanism cannot be demonstrated directly, the chronology strongly supports a role for the restoration of adaptive immunity.

An alternative hypothesis is a cryoablation-induced abscopal effect. The abscopal effect, defined as a systemic antitumor response triggered by local therapy, has predominantly been described in association with radiotherapy [16]. Reports of abscopal effects following cryotherapy remain exceptional, especially in patients not receiving immunotherapy [17]. Cryoablation is known to induce a highly immunogenic form of tumor destruction. Repeated freeze–thaw cycles lead to the release of intact tumor antigens and damage-associated molecular patterns (DAMPs), such as HMGB1, calreticulin, and heat-shock proteins, which can promote dendritic cell activation and antigen presentation. This process can theoretically prime a tumor-specific T-cell response, acting as an in situ vaccine [18]. Cryoablation has been shown to induce immunogenic cell death, leading to antigen release and activation of dendritic cells, thereby promoting systemic antitumor immunity [19]. This phenomenon may contribute to abscopal effects, although such events remain rare in the absence of concomitant immunotherapy [20].

In our case, the regression of multiple pulmonary metastases occurred after cryoablation of a single, symptomatic bone lesion, with no concurrent systemic therapy. This observation is compatible with a cryoablation-mediated systemic immune activation, although the rarity of such events in the absence of immunotherapy makes this hypothesis exceptional [21].

The temporal sequence observed in our patient suggests that these two mechanisms may not be mutually exclusive but rather synergistic. The withdrawal of immunosuppression could have restored immune competence, while cryoablation provided a source of tumor antigens and pro-inflammatory signals necessary to initiate an effective systemic response. The intrinsic immunogenicity of ccRCC may have further amplified this process, facilitating a clinically meaningful antitumor effect.

ccRCC is among the most immunogenic solid tumors, characterized by a high infiltration of immune cells, a rich neoantigenic landscape, and spontaneous immune-related fluctuations in tumor behavior [22]. These features partly explain historical observations of spontaneous metastasis regression and the longstanding sensitivity of RCC to immune-modulating therapies prior to the immunotherapy era. It is therefore plausible that the innate immunogenicity of RCC amplified the impact of immune restoration or local ablative therapies, allowing the immune system to mount an effective antitumor response.

This case has several limitations. First, histological confirmation was only obtained for the pulmonary lesion, while lymph node and bone involvement were inferred based on imaging and clinical evolution. Second, no immunological analyses were performed to directly demonstrate immune reactivation or characterize the tumor microenvironment. Third, the relatively small size of some metastatic lesions may have introduced measurement variability, although the consistency and magnitude of tumor regression across multiple sites and over time argue against a purely radiological artifact.

Despite these limitations, this observation has potential clinical implications. It highlights the importance of considering the impact of immunosuppressive therapies on tumor behavior and raises the possibility that their withdrawal may, in selected cases, restore antitumor immunity. Moreover, it supports the concept that local ablative therapies may have systemic immunomodulatory effects, particularly in immunogenic tumors such as RCC. Further studies are warranted to better characterize these interactions and to identify patients who may benefit from such approaches.

## 4. Conclusions

This case represents an exceptionally rare example of spontaneous regression of mRCC without systemic therapy. While immune restoration following MTX discontinuation is a plausible mechanism, the temporal relationship with cryoablation raises the possibility of an abscopal effect induced by local immunogenic stimuli. The combination of immune reconstitution, ablative therapy, and the intrinsic immunogenicity of ccRCC may have synergistically contributed to the durable partial response observed. This observation underscores the complex interplay between local therapies and systemic antitumor immunity in RCC and highlights the potential of ablative techniques to modulate immune responses, particularly in tumors with strong immunogenic characteristics.

## Data Availability

The original contributions presented in this study are included in the article. Further inquiries can be directed to the corresponding author.
